# Positive contrast spiral imaging for visualization of commercial nitinol guidewires with reduced heating

**DOI:** 10.1186/s12968-015-0219-9

**Published:** 2015-12-22

**Authors:** Adrienne E. Campbell-Washburn, Toby Rogers, Burcu Basar, Merdim Sonmez, Ozgur Kocaturk, Robert J. Lederman, Michael S. Hansen, Anthony Z. Faranesh

**Affiliations:** Cardiovascular and Pulmonary Branch, Division of Intramural Research, National Heart, Lung, and Blood Institute, National Institutes of Health, Bethesda, MD 20892 USA; Institute of Biomedical Engineering, Bogazici University, Istanbul, Turkey

**Keywords:** Interventional CMR, Spiral, Non-Cartesian, Positive contrast, White marker, Guidewire, Real-time

## Abstract

**Background:**

CMR-guidance has the potential to improve tissue visualization during cardiovascular catheterization procedures and to reduce ionizing radiation exposure, but a lack of commercially available CMR guidewires limits widespread adoption. Standard metallic guidewires are considered to be unsafe in CMR due to risks of RF-induced heating. Here, we propose the use of RF-efficient gradient echo (GRE) spiral imaging for reduced guidewire heating (low flip angle, long readout), in combination with positive contrast for guidewire visualization.

**Methods:**

A GRE spiral sequence with 8 interleaves was used for imaging. Positive contrast was achieved using through-slice dephasing such that the guidewire appeared bright and the background signal suppressed. Positive contrast images were interleaved with anatomical images, and real-time image processing was used to produce a color overlay of the guidewire on the anatomy. Temperature was measured with a fiber-optic probe attached to the guidewire in an acrylic gel phantom and in vivo.

**Results:**

Left heart catheterization was performed on swine using the real-time color overlay for procedural guidance with a frame rate of 6.25 frames/second. Using our standard Cartesian real-time imaging (flip angle 60°), temperature increases up to 50 °C (phantom) and 4 °C (in vivo) were observed. In comparison, spiral GRE images (8 interleaves, flip angle 10°) generated negligible heating measuring 0.37 °C (phantom) and 0.06 °C (in vivo).

**Conclusions:**

The ability to use commercial metallic guidewires safely during CMR-guided catheterization could potentially expedite clinical translation of these methods.

**Electronic supplementary material:**

The online version of this article (doi:10.1186/s12968-015-0219-9) contains supplementary material, which is available to authorized users.

## Background

Cardiovascular magnetic resonance (CMR) guidance for cardiovascular catheterization is an appealing alternative to traditional X-Ray guidance, offering improved tissue visualization during procedures and reduced ionizing radiation exposure. Unfortunately, the clinical translation of this promising methodology has been limited by the unavailability of interventional devices that are visible and safe during CMR imaging [1, 2].

The unavailability of guidewires for CMR-guided procedures is especially problematic. Guidewires are used to navigate tortuous anatomy and serve as the rail over which interventional devices such as balloons or stents are delivered, and are thus essential for most catheterization procedures. However, the safety of commercial metallic guidewires remains the primary roadblock for clinical translation of interventional CMR. All metallic guidewires are prone to dangerous levels of RF-induced heating caused by resonating RF waves on the elongated conductor [3–7]. Limited clinical success has been reported using guidewires specifically designed for the CMR environment, to-date. Rather than designing CMR-specific guidewires to possess the required mechanical properties, it would be advantageous to enable the safe use of commercially available guidewires for CMR-guided procedures.

Guidewire visualization is also problematic. Steel guide wires cause severe and unavoidable imaging artifacts that preclude their use. Nitinol guidewires are paramagnetic and create a modest signal void on CMR images, however visualization with this signal void alone is insufficient to safely guide a procedure. Instead, positive contrast methods, where the nitinol guidewire produces a bright signal compared to the background can improve the guidewire conspicuity [8–12]. Previously, we reported the improved visualization of commercial nitinol guidewires using real-time positive contrast with a color overlay of the guidewire on anatomical images [13].

Here, we propose that RF-efficient pulse sequences can be used to relieve concerns of RF-induced heating in standard nitinol guidewires. RF-induced heating (ΔT) follows known dependencies:

1 $$ \varDelta T\propto flip\kern0.5em  angl{e}^2\kern1em \mathrm{and}\kern1em \varDelta T\propto \frac{1}{TR} $$

Thus, spiral imaging, which uses fewer RF pulses, longer readout times and lower flip angles (gradient echo acquisition), is appealing to reduce RF-induced heating. Furthermore, spiral imaging may enable high-frame rate, making it attractive for interventional applications [14].

In this study, we demonstrate the use of commercial nitinol guidewires for cardiovascular catheterization under CMR guidance, with negligible RF-induced heating during spiral gradient echo imaging. In addition, improved real-time guidewire visualization is enabled using positive contrast imaging with color overlay.

## Methods

### Imaging sequence

Imaging was performed on a 1.5 T scanner (Aera, Siemens, Erlangen, Germany). For spiral gradient echo (GRE) imaging, variable density spiral gradient waveforms were calculated within the pulse sequence program using freely available software (http://mrsrl.stanford.edu/~brian/vdspiral/).

**Fig. 1:**
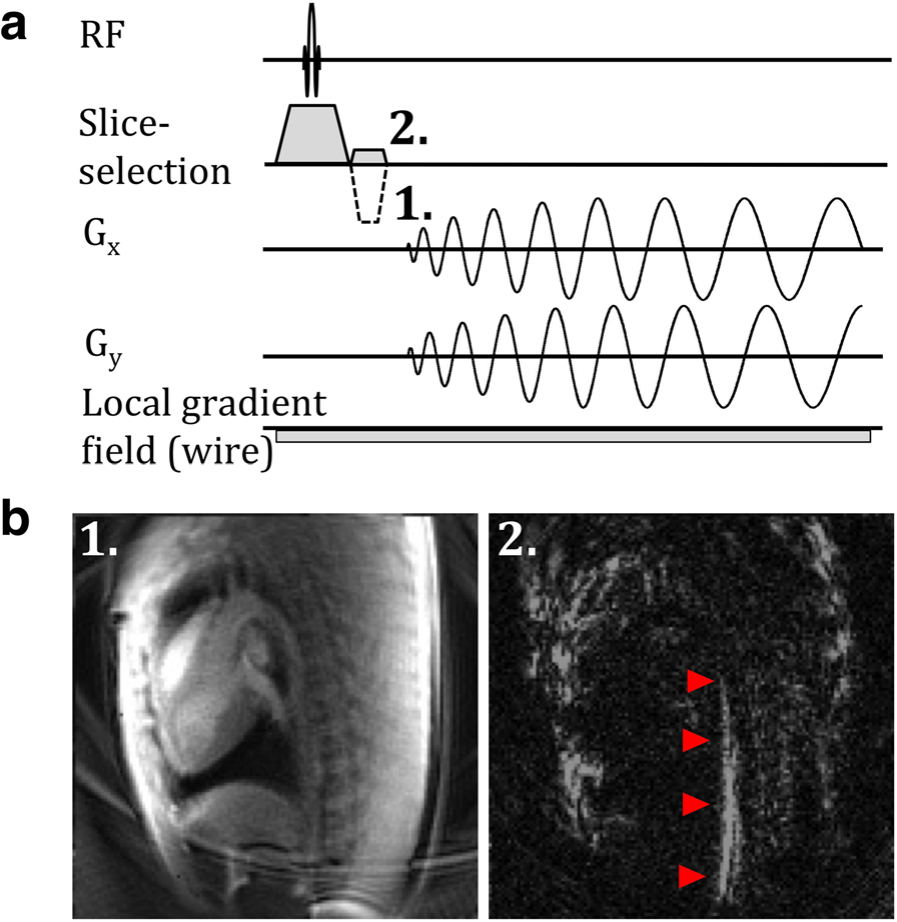
**a** Pulse sequence diagram for spiral gradient echo imaging demonstrating change in slice refocusing gradient to transition from standard anatomical contrast (1) to positive contrast (2). **b** Resulting in vivo anatomical (1) and positive contrast (2) images with red arrowheads indicating nitinol guidewire in the aorta of a Yorkshire swine

Positive contrast of the guidewire was achieved using through-slice dephasing [8, 13]. Figure 1 demonstrates the modified slice-refocusing gradient to generate through-slice dephasing and positive contrast. Using this method, background signal was not fully refocused following slice selection and appeared dark. The local magnetic field gradient created by the guidewire adds to the sequence gradients such that signal surrounding the guidewire is refocused and the guidewire appears bright. A positive contrast guidewire image was interleaved between standard contrast anatomical images.

To correct for image distortions caused by imperfections in the spiral gradient waveforms [15], the gradient impulse response function [16] was measured and used for real time prediction of the true k-space trajectories as previously described [17]. Data was regridded according to these predicted trajectories for image distortion correction. Real-time image reconstruction was performed using open-source reconstruction software (Gadgetron [18], http://gadgetron.github.io) running on an 64-bit (Intel Xeon E7) Linux workstation with 128GB of memory outfitted with a graphics processing unit (NVIDIA Tesla K20Xm) with 5GB of memory. Following image reconstruction, the images were piped back into the Siemens reconstruction environment (Image Calculation Environment, Siemens Healthcare, Erlangen, Germany) for further image processing to isolate guidewire signal and produce the real-time color overlay displayed to the interventionist.

### Real-time color overlay

To assist in procedural guidance, the guidewire signal was extracted from the positive contrast images and overlaid on the anatomical images acquired in alternating frames. To isolate guidewire signal from background contamination caused by other sources of magnetic field inhomogeneity (e.g. air-tissue interfaces), image processing was performed in the Siemens reconstruction environment. The following heuristically-determined image processing steps were performed.

1. Apply Gaussian window to image, in order to suppress signal at the chest wall relative to signal within the vasculature.
2. Apply global threshold to select high intensity pixels [signal intensity > (mean + 1 std image signal intensity)].
3. Apply adaptive local signal intensity threshold to improve guidewire detection. This applies prior knowledge that the high-intensity guidewire signal is mostly surrounded by low-intensity background, whereas other sources of positive contrast create widespread high intensity regions. The guidewire signal has a width of 5–6 pixels, thus we used an 11 × 11 pixel region and selected pixels with [signal intensity > (mean + 1 S.D. local signal intensity)].
4. Find connected regions of pixels.
5. Using prior knowledge that the guidewire is a large elongated structure, keep only regions with eccentricity > 0.95 and # pixels >15.

**Fig. 2:**
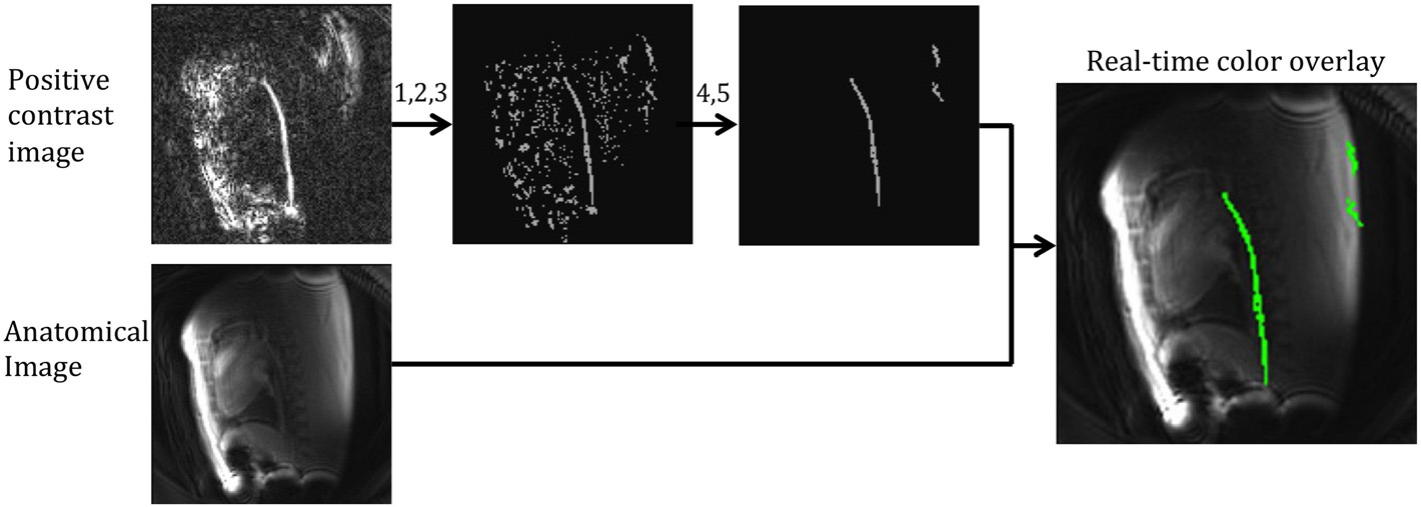
Image processing algorithm used to isolate the guidewire signal from the background in the positive contrast image. Thresholding (Steps 1, 2 and 3) and selection of elongated connected structures (Step 4 and 5) are displayed. The device signal is overlaid in green on the anatomical image and displayed to the interventionist for procedural guidance

The results of these image processing steps are displayed in Fig. 2. The guidewire signal was overlaid in a green color on the anatomical images and displayed on the Siemens Interactive Front End software [19] (Siemens Corporate Research, Princeton, NJ) for procedural guidance.

### Left heart catheterization

The positive contrast spiral sequence, with color overlay, was used to guide left heart catheterization with a 0.035” × 145 cm nitinol guidewire (Nitrex, Covidien, Plymouth, MN) in Yorkshire swine. The guidewire was inserted into the left ventricle from the femoral arterial access site. Animal experiments were conducted with approval from the institutional animal care and use committee according to contemporary NIH guidelines.

Spiral imaging parameters were as follows: 8 interleaves, TE/TR = 0.86/10 ms, flip angle = 10°, slice thickness = 12 mm, FOV = 400 mm, matrix = 128 × 128, BW = 1000 Hz/Px, 24 receive channels. Dephasing in alternating frames was generated using the inverted slice-refocusing gradient with zeroth moment reduced to 25 % of the true slice refocusing moment.

### Phantom guidewire heating measurements

A fiber-optic temperature probe (OpSense, Quebec, Canada) was affixed to a standard commercial nitinol guidewire (0.035” × 145 cm Nitrex, Covidien, Plymouth, MN). The temperature probe was contained within a polymide tube fastened to the guidewire using heat shrink tubing (Advanced Polymers, Salem, NH).

Heating tests were performed in acrylic gel phantom, prepared according to ASTM 2182 (“Measurement of radiofrequency induced heating on or near passive implants during magnetic resonance imaging”) to have a conductivity of 0.26 ± 10 % S/m. The guidewire was aligned parallel to the magnetic field at an off-isocenter position (insertion length = 35 cm, horizontal offset = 12.7 cm, vertical offset = 6 cm). Temperature was measured at the tip of the guidewire during 2 minutes of continuous scanning and compared between 3 different pulse sequences: 1) the proposed spiral GRE, 2) Cartesian GRE and 3) Cartesian balanced stead-state free precession (bSSFP). Cartesian bSSFP was tested because it is the preferred real-time imaging sequence used for clinical MRI-guided right heart catheterizations [20], whereas Cartesian GRE was tested because it uses low flip angles and generates similar contrast to the proposed spiral GRE sequence. Based on the flip angle and TR, it is expected that Cartesian bSSFP will generate the most heating (flip angle = 45° or 60°, TE/TR = 1.31/2.54 ms), Cartesian GRE will generate less (flip angle = 10° or 20°, TE/TR = 1.31/2.86 ms) and spiral GRE will generate negligible heating (flip angle = 10°, 8 interleaves: TE/TR = 0.82/8.52 ms, 16 interleaves: TE/TR = 0.82/5.08 ms). All imaging sequences shared the following parameters: slice thickness = 6 mm, FOV = 300 mm, matrix = 128 × 128.

Three guidewire configurations were compared in the phantom: 1) guidewire alone, 2) guidewire within a nylon-braided 7 F catheter (Goodale-Lubin, Medtronic, Minneapolis, MN) (co-localized distal tips) and 3) guidewire extended 10 cm from catheter distal tip. This catheter was chosen because the lumen was large enough for the guidewire and temperature probe assembly and it contained no metallic components along its length. The guidewire tip was placed at the same off-isocenter position for the three conditions. The temperature increase was calculated from the mean 30 s baseline temperature and mean temperature in final 1 s of scanning. Temperature returned to approximately the same baseline temperature (range: 19.8 °C to 20.9 °C) between each experiment.

Measured temperatures were compared to theoretical predictions according to TR and flip angle dependencies, relative to ΔT_max_ generated by Cartesian bSSFP using flip angle (α) = 60° and TR = 2.54 ms:

2 $$ \varDelta {T}_{theory}=\frac{\alpha^2}{\alpha_{max}^2}\cdot \frac{T{R}_{max}}{TR}\cdot \varDelta {T}_{max} $$

### In vivo guidewire heating measurements

In vivo heating was assessed in a 60 kg Yorkshire swine with femoral arterial vascular access. To compare worst-case and best-case heating, temperature was measured during 2 minutes of continuous scanning with Cartesian bSSFP (flip angle = 45° and 60°, TE/TR = 1.31/2.62 ms) and spiral GRE (flip angle = 10°, 8 interleaves with TE/TR = 0.82/8.23 ms and 16 interleaves with TE/TR = 0.82/4.93 ms). The nitinol guidewire and 6 F catheter were examined at three insertion lengths: 1) guidewire within the introducer sheath, 2) guidewire tip in descending aorta at the level of the diaphragm and 3) guidewire tip at the aortic arch. Because of the stiffness of the guidewire with temperature probe assembly, the guidewire was not inserted into the left-ventricle.

## Results

### Real-time positive contrast spiral imaging

**Fig. 3:**
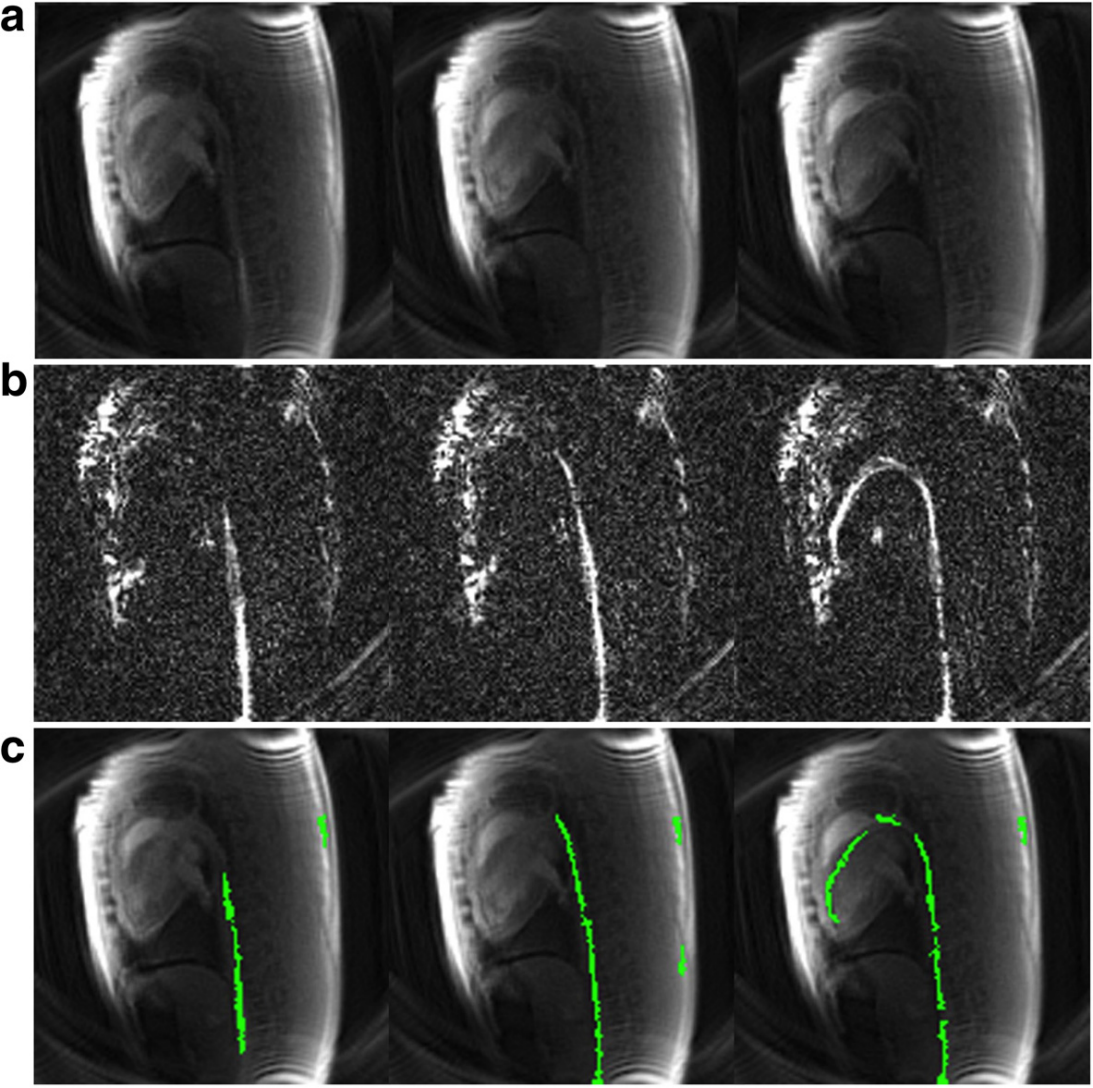
Example left heart catheterization data set showing anatomical images (**a**), positive contrast images (**b**) and resulting color overlay (**c**) for three different insertion lengths: guidewire tip in descending aorta (*left*), guidewire tip at aortic arch (*middle*) and guidewire in left ventricle (*right*)

Through-slice dephasing successfully generated positive contrast from the guidewire in vivo (Fig. 1b). During left heart catheterization, each frame was generated with a temporal resolution of 80 ms (12.5 frames/s), and a pair of positive contrast and anatomical images was generated with a temporal resolution of 160 ms (6.25 frames/s). Reconstruction and image processing occurred in real-time with no latency to produce the color overlay. Figure 3 shows the anatomical image, dephased image and color overlay for three example frames during left heart catheterization. The guidewire was easily discernable in the positive contrast images at all locations during the left-heart catheterization. Additional file 1 provides a video of the raw positive contrast images from a swine undergoing left heart catheterization with the commercial nitinol guidewire, and Additional file 2 shows the color overlay seen by the interventionist (online material).

### Guidewire heating

The temperature was measured along the length of the guidewire to confirm that the hotspot of the guidewire was located at the guidewire distal tip, as theoretically predicted. As expected, the Cartesian bSSFP sequence with flip angle = 60° generated the most heating because of the short TR and high flip angle. The 16 interleave spiral sequence generated more heating than the 8 interleave sequence because of the shorter TR.

**Fig. 4:**
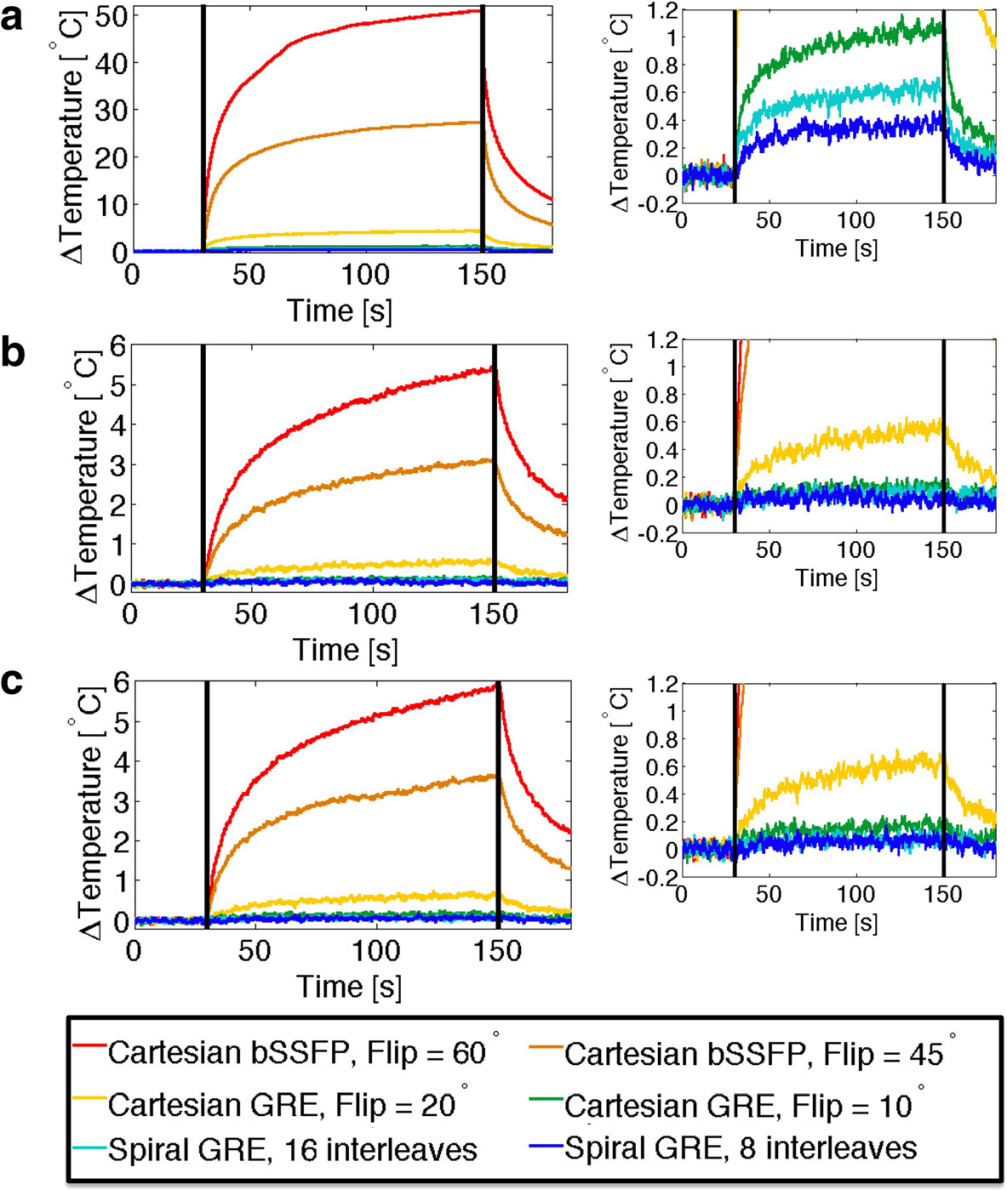
Temperature increase observed in an acrylic gel phantom during 2 minutes of continuous scanning (scanning period indicated by *vertical black lines*). Temperature is measured at the tip of the nitinol guidewire for the guidewire and catheter co-located (**a**), guidewire extended 10 cm distal to catheter (**b**) and guidewire alone (**c**). Right-hand plots show reduced temperature scale (−0.2 °C to 1.2 °C ) to demonstrate subtle heating during GRE imaging

Figure 4 shows the recorded temperature during 2 minutes of continuous scanning in the ASTM gel phantom. Maximal heating was observed with the guidewire and catheter assembly (tips co-localized). Lesser heating was observed with the other two configurations: guidewire alone and guidewire extended 10 cm from catheter. Negligible heating (ΔT <0.7 °C) was observed in the acrylic gel phantom using spiral GRE imaging with 8 or 16 interleaves during 2 minutes of scanning for all guidewire configurations. Cartesian GRE also generated very little heating in all configurations (ΔT < 1.1 °C for flip angle = 10°; ΔT < 4.5 °C for flip angle = 20°). In comparison, our standard real-time imaging sequence, Cartesian bSSFP, generated substantial heating in the guidewire, as much as 50.4 °C (for flip angle = 60°). Table 1 provides the maximum temperature increase from baseline for each guidewire configuration and imaging sequence. Table 1 also demonstrates that these values match theoretical predictions calculated by Eqn 2. Note that the TempSens Signal conditioner (OpSense, Quebec, Canada) has an accuracy of ± 0.3 °C, and baseline temperature measurements fluctuated with a standard deviation of ± 0.03 °C.

**Table 1:** Nitinol guidewire heating in ASTM gel phantom

	Wire + Catheter		Wire 10 cm distal to catheter		Wire only	
	Measured	Theoretical	Measured	Theoretical	Measured	Theoretical
Cartesian bSSFP (flip = 60°, TR = 2.54 ms)	50.39 °C	--	5.41 °C	--	5.90 °C	--
Cartesian bSSFP (flip = 45°, TR = 2.54 ms)	27.37 °C	28.34 °C	3.10 °C	3.05 °C	3.05 °C	3.32 °C
Cartesian GRE (flip = 20°, TR = 2.86 ms)	4.31 °C	4.97 °C	0.56 °C	0.53 °C	0.65 °C	0.58 °C
Cartesian GRE (flip = 10°, TR = 2.86 ms)	1.07 °C	1.24 °C	0.14 °C	0.13 °C	0.16 °C	0.15 °C
Spiral GRE (16 interleaves, flip = 10°, TR = 5.16 ms)	0.61 °C	0.70 °C	0.09 °C	0.08 °C	0.08 °C	0.08 °C
Spiral GRE (8 interleaves, flip = 10°, TR = 8.60 ms)	0.37 °C	0.44 °C	0.03 °C	0.05 °C	0.07 °C	0.05 °C

Temperature increase from baseline observed during 2 minutes of continuous imaging in an acrylic gel phantom. Temperature was measured at the tip of the nitinol guidewire (Nitrex, Covidien, Plymouth, MN) with the guidewire collocated with the catheter, with the guidewire extended 10 cm distal to catheter and with the wire alone. Theoretical predications, based on flip angle and TR (Eqn2), of the relative heating compared to Cartesian bSSFP (flip angle = 60°) are also displayed

**Fig. 5:**
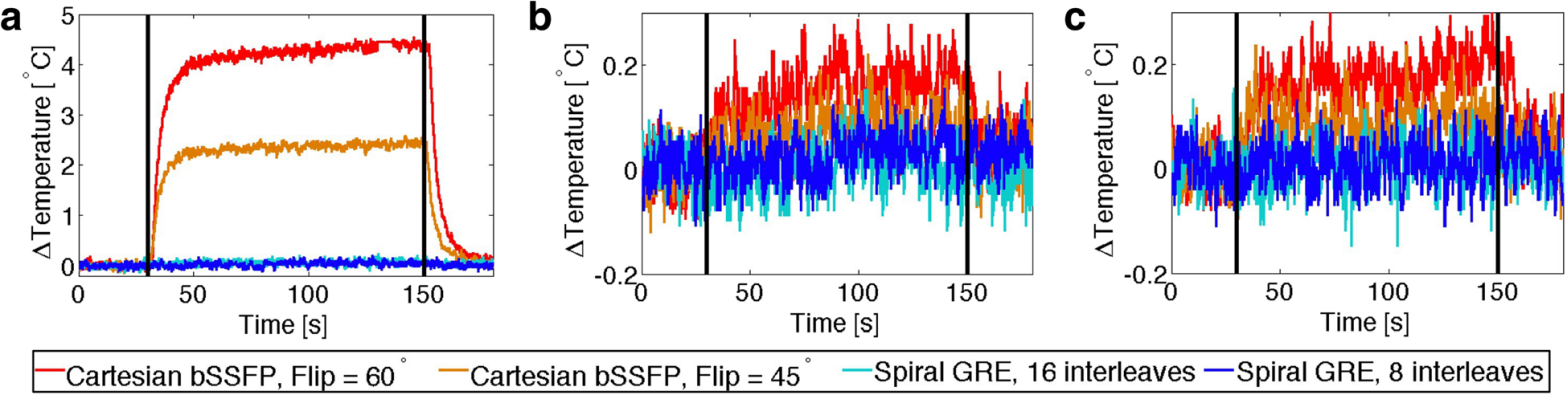
In vivo temperature measurements at the tip of the nitinol guidewire and catheter at three insertion lengths: guidewire tip within the introducer sheath (**a**), in the descending aorta (**b**) and at the aortic arch (**c**)

Much less heating was expected in vivo due to blood flow cooling. Figure 5 shows the recorded temperature at the tip of the guidewire during in vivo imaging and the maximum temperatures observed are provided in Table 2 for each guidewire configuration and anatomical location. Worst-case heating was observed when the guidewire was still contained within the introducer sheath, because of the insulation provided by the sheath and the far off-isocenter position. Furthermore, observable heating was generated using Cartesian bSSFP imaging within the vasculature. No heating was observed in vivo using spiral GRE under any conditions. Throughout the course of the in vivo heating experiments (~3 hours), the core temperature of the animal was raised by 1 °C, as measured by a rectal temperature probe.

**Table 2:** Nitinol guidewire heating in vivo

	Wire + Catheter			Wire 10 cm distal to catheter		
	Sheath	Aorta	Arch	Sheath	Aorta	Arch
Cartesian bSSFP (flip = 60°, TR = 2.86 ms)	4.34 °C	0.18 °C	0.14 °C	0.22 °C	0.13 °C	0.05 °C
Cartesian bSSFP (flip = 45°, TR = 2.86 ms)	2.50 °C	0.08 °C	0.10 °C	0.17 °C	0.09 °C	0.04 °C
Spiral GRE (16 interleaves, flip = 10°, TR = 5.16 ms)	0.09 °C	−0.04 °C	0.01 °C	−0.02 °C	−0.02 °C	0.00 °C
Spiral GRE (8 interleaves, flip = 10°, TR = 8.60 ms)	0.06 °C	0.04 °C	0.06 °C	0.00 °C	0.03 °C	0.03 °C

In vivo temperature increase from baseline observed during 2 minutes of continuous imaging measured at the tip of the nitinol guidewire. Temperature was measured with the guidewire tip in the introducer sheath, in the aorta and at the aortic arch in a 60 kg swine

## Discussion

### Positive contrast spiral imaging

Here, we have demonstrated spiral GRE imaging for substantially reduced heating of a commercial nitinol guidewire in the scanner. In addition, using positive contrast imaging with real-time color overlay, we have demonstrated improved passive visualization of a commercial nitinol guidewire. Existing nitinol guidewires have a range of mechanical properties (stiffness, curvature, torquability, surface coating, geometry) to address procedural needs, and it would be beneficial to use these existing products for CMR-guided procedures. Thus, the ability to safely and effectively use a commercial nitinol guidewire in the CMR environment could accelerate the clinical translation of CMR-guided cardiovascular catheterization procedures.

Positive contrast spiral imaging was achieved using through-slice dephasing. Positive contrast was observed at a range of dephasing gradient moments, indicating that the choice of desphasing moment does not have to be precisely tuned [13]. Here, for left-heart catheterization experiments, the slice-refocusing gradient was inverted and reduced the zeroth moment to 25 %. This value was chosen to eliminate most background signal, while maintaining signal the entire guidewire length. Separate anatomical and guidewire images were acquired in an interleaved fashion, reducing the achievable frame rate to 6.25 frames/s using the sequence parameters presented here. This frame rate is equivalent to what is used for CMR-guided right heart catheterization in patients with accelerated bSSFP imaging [20]. Future work could investigate parallel imaging for improved frame rate or alternate acquisition schemes with the device image updating more frequently than the anatomical image. Reasonable image quality was also observed for anatomical imaging.

Positive contrast has been used for both interventional device imaging and cell tracking using iron oxide nanoparticles [8–13, 21–24]. Positive contrast has been achieved using dephasing in the slice-encoding and frequency-encoding directions, using on-resonance signal suppression, exploiting the off-resonance properties of bSSFP and with susceptibility gradient mapping in post-processing. Dephasing-based methods are the most conducive to real-time imaging. To our knowledge, this is the first time that positive contrast methods have been combined with spiral imaging, with the goal of reducing RF-induced heating. Here, because we are using a GRE sequence [10, 12], all positive contrast signal is a result of local magnetic field gradients counteracting the dephasing gradient.

### Real-time color overlay

The real-time color overlay used here permits intuitive visualization of the guidewire, mimicking active device visualization. We have demonstrated similar visualization methods with a dual echo bSSFP sequence previously [13]. The positive contrast device imaging is non-specific, and other regions of magnetic field inhomogeneity (e.g. air-tissue interfaces) also produce bright signal. Image processing can isolate guidewire signal reliably in most configurations. The air-tissue interface of the lung at the level of the aortic arch is closely neighboring the vasculature, and therefore creates the most challenging region to isolate guidewire signal. Also, the tapered tip of the guidewire is problematic, as the positive contrast signal is reduced at the guidewire tip. Thus, the color overlay is least reliable as the guidewire tip curves around the aortic arch. Additional color overlay artifacts result from background positive contrast signal matching the large/elongated structure criteria. These image processing artifacts mainly appear outside of the vasculature and therefore are not distracting to interventionists performing the procedure.

Furthermore, the guidewire can move out of plane, reducing reliability of color overlay. A thick slice of 12 mm was used for left-heart catheterization to avoid the guidewire moving out of plane during imaging.

### Guidewire heating

Heating tests were performed in an acrylic gel phantom, where heat dissipation is minimal, in order to generate worst-case scenario temperature increases. Furthermore, due to increased insulation, heating was found to be maximal when the guidewire was used in combination with a catheter with the tips co-localized. Although this configuration would not be used clinically for an entire procedure, it is plausible that the guidewire and catheter tips would coincide at some point during navigation, and thus it was used to assess worst-case scenario heating in vivo.

The safety improvements enabled by spiral GRE are clearly illustrated from the phantom experiments. In the gel phantom, unsafe levels of heating (50 °C ) were observed using Cartesian bSSFP with standard parameters. In direct comparison, using spiral GRE imaging, temperature increases remained <0.7 °C. Cartesian GRE with 10° flip angle also reduces heating substantially (<1.1 °C), however we have focused on spiral imaging because of the high frame-rate and SNR-efficiency.

Heating regulations have been established for other medical devices. International standard for MRI safety IEC 60601-2-33:2008 [25] sets a body temperature threshold of 39 °C during non-invasive imaging. Additionally, IEC 60601–1:2005 [26] section 11.1.2.2 covers invasive devices and sets a body temperature threshold of 43 °C for continuous exposure and proposes “Where 41 °C is not exceeded, no justification is required”. In compliance with this regulation, marketed intracameral ultrasound probes (e.g. Siemens Acuson Acunav 8 Fr intracardiac ultrasound catheter [27]) will suspend operation if the temperature reaches 43 °C.

In a 60 kg swine, we observed a localized increase of 4 °C above body temperature (local temperature ~41 °C) using standard real-time Cartesian bSSFP imaging, emphasizing the potential safety concerns of metallic guidewire imaging in humans. In comparison, no heating was observed using spiral GRE imaging, indicating the potential safety benefit of moving to RF-efficient sequences.

Heating is very sensitive to a wide variety of factors such as off-isocenter position, guidewire length, insertion length and curvature [3–6]. Here, only a small subset of possible conditions were tested, and further extensive heating tests are required to validate the safety of spiral GRE under all possible configurations used during catheterization procedures.

### Limitations

This method is limited by the non-specific nature of the positive contrast signal. This results in less reliable color overlay of the device signal due to errors in image processing. Future work will investigate alternative image processing methods to improve the specific isolation of the guidewire signal. Furthermore, because there is no unique guidewire tip signal, it is impossible to determine if the tip of the guidewire is contained within any given image. Future work will investigate unique tip markers for passive guidewires, as well as automated slice repositioning to maintain the guidewire tip within the imaging frame [28]. In general, gradient echo imaging has reduced blood-myocardium contrast compared to the bSSFP sequence typically used for real-time imaging [29]. Spiral imaging is also susceptible to off-resonance blurring and image distortion, which has been corrected here using a real-time framework [17]. Despite the reduced blood-myocardium contrast and possible spiral imaging artifacts, the inherent safety of spiral GRE imaging makes this tradeoff worthwhile.

## Conclusion

RF-efficient spiral GRE imaging is valuable to reduce heating of nitinol guidewires under CMR to a negligible level. Spiral GRE can be combined with positive contrast imaging to improve visualization of the guidewire in vivo. The results presented here are promising to permit the use of commercial nitinol guidewires safely and effectively for CMR-guided catheterizations.

## Additional files

Additional file 1: 
**Raw positive contrast spiral GRE images used for guidewire visualization during CMR-guided left heart catheterization of a swine.** (MP4 3563 kb) Additional file 2:
**Real-time guidewire color overlay seen by the interventionist during CMR-guided left heart catheterization of a swine generated from interleaved anatomical and positive contrast spiral GRE images.** (MP4 1636 kb)

## Abbreviations

bSSFP: balanced steady state free precession
BW: bandwidth
FOV: field of view
GRE: Gradient Recalled Echo
ICE: Image calculation environment
IEC: International Electrotechnical Commission
IFE: Interactive front end
RF: radiofrequency
TE: echo time
TR: repetition time
